# Gut, oral and nasal microbiota and Parkinson’s disease

**DOI:** 10.1186/s12934-020-01313-4

**Published:** 2020-02-27

**Authors:** Liang Shen

**Affiliations:** 1 Institute of Biomedical Research, Shandong University of Technology, Zibo, Shandong People’s Republic of China; 2 Shandong Provincial Research Center for Bioinformatic Engineering and Technique, Zibo Key Laboratory of New Drug Development of Neurodegenerative Diseases, School of Life Sciences, Shandong University of Technology, Zibo, Shandong People’s Republic of China

**Keywords:** Parkinson’s disease, Gut microbiota, Oral microbiota, Nasal microbiota

## Abstract

Parkinson’s disease (PD) is the second most prevalent neurodegenerative disease, and in an effort to identify novel therapeutic target for this disease in recent years, human microbiota has attracted much interest. This paper briefly summarizes the main findings concerning the differences of human microbiome across several important mucosal interfaces, including nose, mouth, and gut between PD patients and controls as obtained from a total of 13 studies published since 2015, which covered a total of 943 PD patients and 831 matched controls from 6 countries. Overall, these studies supported the differences of gut microbiota between PD patients and matched controls, while significantly altered bacterial taxa among studies were not identical. Due to relatively limited number of available studies and covered patients, the associations between oral and nasal microbiota and PD remain inconclusive. The therapeutic and diagnostic potentials of gut microbiota for PD are discussed. More well-designed clinical studies recruiting large-scale PD patients are encouraged in future.

## Introduction

Parkinson’s disease (PD) is the second most prevalent neurodegenerative disease in the elderly after Alzheimer’s disease. PD is estimated to affect about 1% of populations over the age of 60 [1]. Clinically, PD is characterized primarily by severe and progressing tremors, rigidity, posture instability, and cognitive impairment. Neuropathologically, the hallmarks of PD mainly include the progressive degeneration of dopaminergic nigrostriatal neurons and the formation of aggregated α-synuclein, called Lewy bodies, in the brain [2, 3]. Despite much progress has been made to understand the genetic and environmental factors contributing to PD development in the past decades, the pathogenesis of PD remains far to be fully elucidated [4, 5]. Currently, available drugs for PD are symptomatic, and disease progression is inexorable, and patients will ultimately suffer from disability. As a result, identifying novel targets to develop new agents to combat PD has gained much interest.

In recent years, the association between human gut microbiota, which comprises approximately 10^14^ microbes, and PD development has sparked increasing attentions [5–9]. Gut microbiota has been proposed to be a potential therapeutic target and also has diagnostic biomarker potential. In addition, as nasal and oral cavities constitute two important ports of entry for a possible pathogenic agent spreading to the central nervous system, which may be involved in the pathogenesis of PD, the oral and nasal microbiota of PD patients have been investigated [10–12]. This paper summarizes the recent literature on the differences of human microbiome across several important mucosal interfaces, including nose, mouth, and gut, between PD patients and controls, and their therapeutic and diagnostic potentials.

## Summary of included studies

**Fig. 1:**
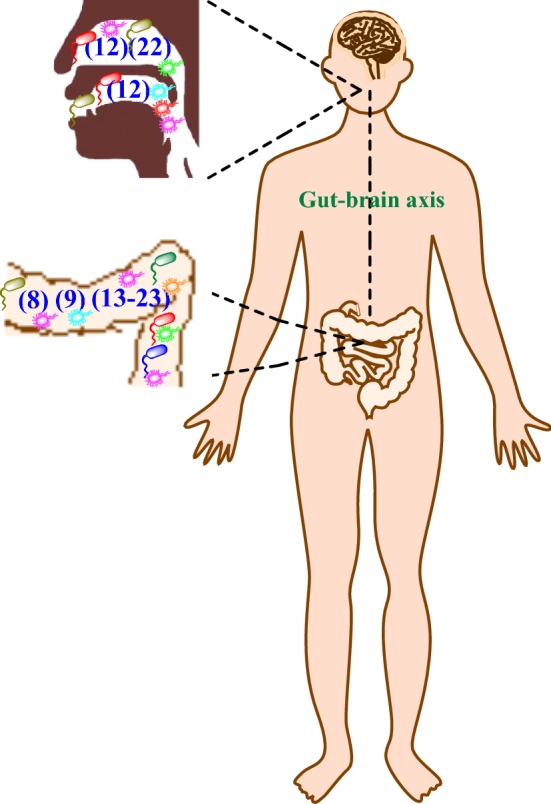
Schematic illustration of the association between nasal, oral and gut microbiota and PD. The number in pathogenesis represents reference order

Through retrieving the PubMed database, a total of 13 eligible studies, which were published between 2015 and 2018, are included in the present review (Table 1). The 13 studies were conducted in 6 countries (4 studies from Germany, 3 from China, 3 from USA, 2 from Finland, 1 from Russia and 1 from Japan, respectively). These studies cover 943 PD patients and 831 controls. The samples range from 38 to 327 cases and controls combined. The 13 studies report 16 sets of microbiota data, 13 are about gut microbiota, 2 are about nasal microbiota, and 1 is about oral microbiota (Fig. 1).

**Table 1:** Gut, oral and nasal microbiota associations with PD

Organs	References	Publication year	Country	Number of patients	Number of controls	Microbiota associations
Gut	Scheperjans et al. [8]	2015	Finland	72	72	Gut microbiota was altered in PD patients and a significant reduction of the relative abundance of *Prevotellaceae* in PD patients in comparison with controls was observed. The relative abundance of *Enterobacteriaceae* was identified to be positively associated with the severity of postural instability and gait difficulty
Gut	Keshavarzian et al. [13]	2015	USA	38	34	The fecal microbiota of PD patients was significantly different from the control subjects. The relative abundances of some butyrate-producing bacteria from the genera Blautia, Coprococcus, and Roseburia were significantly higher in the feces of controls than PD patients
Gut	Hasegawa et al. [14]	2015	Japan	52	36	Abundances of *Clostridium coccoides* and *Bacteroides fragilis* decreased, while that of *Lactobacillus* increased in PD patients than controls
Gut	Unger et al. [9]	2016	Germany	34	34	PD patients possessed decreased abundance of bacterial phylum *Bacteroidetes* and the bacterial family *Prevotellaceae*, while increased abundance of *Enterobacteriaceae* in comparison with controls
Gut	Li et al. [15]	2017	China	24	14	Relative abundances of *Blautia*, *Faecalibacterium* and *Ruminococcus* significantly decreased, and those of *Escherichia*-*Shigella*, *Streptococcus*, *Proteus*, and *Enterococcus* significantly increased in PD subjects compared with controls
Gut	Hopfner et al. [16]	2017	Germany	29	29	There was significant difference in beta diversity indices between PD patients and controls, while not for alpha diversity indices. The abundances of *Lactobacillaceae*, *Barnesiellaceae* and *Enterococcacea* were found to be higher in patients than in controls
Gut	Bedarf et al. [17]	2017	Germany	31	28	Significant difference was observed for the gut microbiota composition between PD patients and controls at all taxonomic levels. PD patients have increased abundances of *Verrucomicrobiaceae* (*Akkermansia muciniphila*) and unclassified Firmicutes, while decreased abundances of *Prevotellaceae* (*Prevotella copri*) and *Erysipelotrichaceae* (*Eubacterium biforme*)
Gut	Hill-Burns et al. [18]	2017	USA	197	130	Significantly differed abundances of *Bifidobacteriaceae*, *Christensenellaceae*, [*Tissierellaceae*], *Lachnospiraceae*, *Lactobacillaceae*, *Pasteurellaceae* and *Verrucomicrobiaceae* families between PD patients and controls were observed
Gut	Petrov et al. [19]	2017	Russia	89	66	Reduced gut microbiota diversity in PD patients was observed. Decreased abundances of *Dorea*, *Bacteroides*, *Prevotella*, *Faecalibacterium*, *Bacteroides massiliensis*, *Stoquefichus massiliensis*, *Bacteroides coprocola*, *Blautia glucerasea*, *Dorea longicatena*, *Bacteroides dorei*, *Bacteroides plebeus*, *Prevotella copri*, *Coprococcus eutactus*, *and Ruminococcus callidus*, *and increased abundances of Christensenella*, *Catabacter*, *Lactobacillus*, *Oscillospira*, *Bifidobacterium*, *Christensenella minuta*, *Catabacter hongkongensis*, *Lactobacillus mucosae*, *Ruminococcus bromii*, and *Papillibacter cinnamivorans*, in PD patients in comparison with controls
Gut	Qian et al. [20]	2018	China	45	45	The richness and diversity of gut microbiota in PD patients were significantly higher compared with healthy group. Several enriched genera were identified in the feces of PD patients, which include *Clostridium IV*, *Aquabacterium*, *Holdemania*, *Sphingomonas*, *Clostridium XVIII*, *Butyricicoccus* and *Anaerotruncus*. The genera *Escherichia/Shigella* were negatively associated with disease duration
Gut	Lin et al. [21]	2018	China	75	45	Significantly increased abundances of four bacterial families and decreased abundances of seventeen bacterial families in PD patients in comparison with controls were observed
Gut	Heintz-Buschart et al. [22]	2018	Germany	76	78	Relative abundances of *Akkermansia* sp. and *Prevotella* sp. were significantly higher in gut microbiota of PD in comparison with healthy controls
Gut	Tetz et al. [23]	2018	USA	31	28	Different microbiota richness and diversity between PD and control groups were observed. A depletion of *Prevotellaceae* and *Lachnospiraceae* and decreased abundances of *Lactobacillaceae* and *Streptococcaceae* in the PD group compared with the controls were observed
Mouth	Pereira et al. [12]	2017	Finland	72	76	Different beta diversity of oral microbiota was found between PD patients and controls. Abundances of *Prevotella*, *Prevotellaceae*, *Veillonella*, *Solobacterium*, *Veillonellaceae*, *Lactobacillaceae*, and *Coriobacteriaceae* increased, while those of *Capnocytophaga*, *Rothia*, *Kingella*, *Leptotrichia*, *Actinomyces*, and *Leptotrichiaceae* decreased, in oral microbiota of PD patients compared with controls
Nose	Pereira et al. [12]	2017	Finland	69	67	No alpha or beta differences between nasal microbiota of control and PD patients were found
Nose	Heintz-Buschart et al. [22]	2018	Germany	76	78	The nasal microbiota displayed higher variation over the different individuals and no significant differences were found between PD patients and controls

## Gut microbiota

There are a total of 13 studies focusing on the gut microbiota of 726 PD patients. The first study was conducted by Scheperjans et al. which compared the gut microbiota of 72 Finnish PD patients and 72 controls by means of 16S rRNA gene amplicon sequencing [8]. The altered gut microbiota of PD patients was demonstrated. A significant reduction by 77.6% of the relative abundance of *Prevotellaceae* in the feces of PD patients compared with controls, and the relative abundance of *Enterobacteriaceae* was identified to be positively associated with the severity of postural instability and gait difficulty [8]. Keshavarzian et al. investigated the colonic bacterial composition of 38 American PD patients and 34 controls by means of 16S rRNA gene amplicon sequencing [13]. Significant difference was observed between the fecal microbiota of PD patients and controls. Further analysis indicated significantly higher relative abundances of some butyrate-producing bacteria, which included genera *Blautia*, *Coprococcus* and *Roseburia*, in the feces of controls in comparison with PD patients [13]. Hasegawa et al. analyzed the gut microbiota of 52 Japanese PD patients and compared with those of 36 controls by employing rRNA-targeted reverse transcription-quantitative PCR [14]. It was found that the abundances of *Clostridium coccoides* and *Bacteroides fragilis* decreased, while that of *Lactobacillus* increased in the gut microbiota of PD patients in comparison with controls. In 2016, Unger et al. reported a comparative analysis of the gut microbiota of 34 German PD patients and 34 age-matched controls by means of quantitative PCR [9]. It was indicated that the abundances of *Bacteroidetes* and *Prevotellaceae* decreased, while that of *Enterobacteriaceae* was enriched in the gut microbiota of PD patients in comparison with controls. In 2017, Li et al. conducted a comparative analysis of the gut microbiota of 24 Chinese patients and 14 healthy controls by 16S rRNA gene amplicon sequencing [15]. It was revealed that the relative abundances of cellulose degraders like *Blautia*, *Faecalibacterium* and *Ruminococcus* significantly decreased, and those of pathobionts, including *Escherichia*-*Shigella*, *Streptococcus*, *Proteus*, and *Enterococcus*, significantly increased in PD subjects compared with healthy controls. Hopfner et al. analyzed the gut microbiota of 29 German PD patients and 29 age-matched controls by 16S rRNA gene amplicon sequencing [16]. It was reported that there was significant difference in beta diversity indices between PD patients and controls, while no significant difference was observed for alpha diversity indices. *Lactobacillaceae*, *Barnesiellaceae* and *Enterococcacea* were found to be more abundant in patients than in controls. The gut microbiota analysis by Bedarf et al. covered 31 early stage, l-DOPA-naïve PD patients and 28 age-matched controls from Germany. Significant difference was observed for the gut microbiota composition between PD patients and controls at all taxonomic levels [17]. It was indicated that PD patients possessed increased abundances of *Errucomicrobiaceae* (*Akkermansia muciniphila*) and unclassified Firmicutes, while reduced abundances of *Prevotellaceae* (*Prevotella copri*) and Erysipelotrichaceae (*Eubacterium biforme*). In the study of Hill-Burns et al. relatively larger samples (197 American PD patients and 130 controls) were covered [18]. By 16S rRNA gene amplicon sequencing, it was found that the abundances of *Bifidobacteriaceae*, *Christensenellaceae*, [*Tissierellaceae*], *Lachnospiraceae*, *Lactobacillaceae*, *Pasteurellaceae and Verrucomicrobiaceae* differed significantly between PD patients and controls. Petrov et al. compared the gut microbiota of 89 Russian PD patients and 66 controls by means of 16S rRNA gene amplicon sequencing [19]. It was indicated that the gut microbiota diversity reduced in PD patients in comparison with controls. The abundances of 14 bacterial taxa *Dorea*, *Bacteroides*, *Prevotella*, *Faecalibacterium*, *Bacteroides massiliensis*, *Stoquefichus massiliensis*, *Bacteroides coprocola*, *Blautia glucerasea*, *Dorea longicatena*, *Bacteroides dorei*, *Bacteroides plebeus*, *Prevotella copri*, *Coprococcus eutactus*, and *Ruminococcus callidus* decreased, while those of *Christensenella*, *Catabacter*, *Lactobacillus*, *Oscillospira*, *Bifidobacterium*, *Christensenella minuta*, *Catabacter hongkongensis*, *Lactobacillus mucosae*, *Ruminococcus bromii*, and *Papillibacter cinnamivorans* increased in PD patients in comparison with controls. In 2018, Qian et al. investigated the differences in gut microbiota between 45 Chinese PD patients and their healthy spouses by means of 16S rRNA gene amplicon sequencing [20]. It was indicated that the richness and diversity of the gut microbiota in PD patients were significantly higher compared with those of control group. Several enriched genera were identified in the feces of PD patients, which include *Clostridium IV*, *Aquabacterium*, *Holdemania*, *Sphingomonas*, *Clostridium XVIII*, *Butyricicoccus* and *Anaerotruncus*. The genera *Escherichia/Shigella* were found to be negatively associated with disease duration. Lin et al. investigated the gut microbiota of 75 Chinese PD patients and 45 age-matched controls by means of 16S rRNA gene amplicon sequencing [21]. It was found the alpha and beta diversity between PD patients and controls did not differ significantly. The abundances of four bacterial families significantly increased and those of seventeen ones decreased in PD patients in comparison with controls. Heintz-Buschart et al. compared the gut microbiota of 76 PD patients and 78 matched healthy individuals by means of 16S and 18S rRNA gene amplicon sequencing [22]. They demonstrated that PD patients possessed significantly increased abundance of *Akkermansia* sp. and *Prevotella* sp. in gut microbiota compared with healthy controls. In addition, Tetz et al. analyzed the gut microbiota of 31 American PD patients and 28 controls by means of shotgun metagenomics sequencing [23]. A depletion of *Prevotellaceae* and *Lachnospiraceae* and decreased abundances of *Lactobacillaceae* and *Streptococcaceae* in the feces of PD patients in comparison with the controls were found.

## Oral and nasal microbiota

The oral and nasal microbiota of PD patients has gained growing attentions and been investigated by three studies. Pereira et al. investigated the oral microbiota of 72 Finnish PD patients and 76 controls employing 16S rRNA gene amplicon sequencing [12]. Through comparative analysis, significant difference in beta diversity of oral microbiota was found between PD patients and control groups. Further analysis identified the increased abundances of *Prevotella*, *Prevotellaceae*, *Veillonella*, *Solobacterium*, *Veillonellaceae*, *Lactobacillaceae*, and *Coriobacteriaceae*, and decreased abundances of *Capnocytophaga*, *Rothia*, *Kingella*, *Leptotrichia*, *Actinomyces*, and *Leptotrichiaceae*, in oral microbiota of PD patients compared with controls.

As to nasal microbiota, the study of Pereira et al. also compared the nasal microbiota of 69 PD patients and 67 controls using 16S rRNA gene amplicon sequencing [12]. It was indicated that no alpha or beta differences existed between the nasal microbiota of PD patients and control groups. In addition, through 16S and 18S rRNA gene amplicon sequencing, Heintz-Buschart et al. analyzed the microbiota of nasal wash samples from 76 Russian PD patients and 78 matched healthy controls, and they found no strong differences in nasal microbiota between PD patients and controls [22].

## Conclusions and perspectives

The associations between PD and human microbiome across several important mucosal interfaces, including nose, mouth, and gut, have sparked much interest in recent years [23–26]. According to the above discussion, the currently available studies support the alterations in gut microbiota in PD patients compared with controls. Nevertheless, despite a few significantly differed bacterial taxa are common in selected studies, the altered bacterial taxa reported in each study was not completely consistent overall. This may derive from the facts that these studies differed in PD patient inclusion criteria, severity of disease, sequencing methodologies, and the treatment of confounders. As to oral microbiota, there is only one study on this issue and found differed beta diversity and some bacterial taxa between patients and controls. Two studies have explored the nasal microbiota, and both indicated no obvious differences in nasal microbiota between PD patients and controls. However, currently, we cannot give an affirmative and negative conclusion concerning the association between oral/nasal microbiota and PD due to the rather limited number of studies and patients.

There should be multiple molecular mechanisms underlying the association between gut microbiota and PD. As one main metabolic product of gut bacteria, the concentrations of short chain fatty acids (SCFAs) were observed to be altered accompanied by altered gut microbiota composition in several studies. Several studies have found less SCFA butyrate-producing bacteria in the feces of PD patients [9, 13], while it has been suggested that decreased levels of SCFAs might decrease colonic motility, and also elevate the gut barrier leakiness [27, 28]. In addition, several studies have identified the decreased abundance of *Prevotellaceae* [8, 9, 17, 19, 23]. It was inferred that decreased *Prevotellaceae* levels could decrease mucin synthesis, and resulted in increased gut permeability. Exposure to bacterial endotoxin (e.g., lipopolysaccharide) caused by increased gut permeability could induce excessive expression and aggregation of α-synuclein, which is crucial in PD development [29–31].

Several animal studies have provided further insights into the association between gut microbiota dysbiosis and pathogenesis of PD. Employing the α-synuclein overexpressing mice model of PD, Sampson et al. found the important role of gut microbiota for motor deficits, microglia activation, and α-synuclein pathology [24]. This was supported by the interesting findings that oral gavaging with specific microbial metabolites to germ-free mice promoted neuroinflammation and motor symptoms, and colonization with microbiota from PD patients could enhance physical impairments in α-synuclein-overexpressing mice [24]. Yang et al. reported that oral administration of rotenone led to gastrointestinal dysfunction and microbiome dysbiosis prior to motor dysfunction of mice model of PD induced by rotenone, and gut microbiota dysbiosis might contribute to rotenone toxicity in PD initiation [26]. Similarly, Perez-Pardo et al. also revealed that the gut microbiota of mice model of PD induced by rotenone was characterized by a significant decrease in the relative abundance of the genus *Bifidobacterium*, and gut microbiota dysbiosis might play an important role in the disruption of intestinal epithelial integrity as well as intestinal inflammation, which are potentially associated with PD pathology [25].

The following aspects may deserve attentions in future studies. First, most studies discussed above employed 16S rRNA gene amplicon sequencing, and only one used the shotgun metagenomics sequencing during gut microbiota analysis. As we know, 16S rRNA gene amplicon sequencing may be biased owing to unequal amplification of species’ 16S rRNA genes, and is not deep enough to detect all species. Second, the number of samples in some included studies are relatively small, and the inclusion criteria of PD patients varies among the included studies. Thus, more studies recruiting large-scale patients with new generation sequencing methodology, are encouraged to investigate the association between human microbiota and PD. Third, several clinical studies have indicated the benefits of supplying probiotics or in combination with prebiotics for PD [32, 33]. For instance, Barichella et al. conducted a randomized, double-blind, placebo-controlled trial and it was found that consumption of fermented milk containing probiotics and prebiotics could increase the frequency of complete bowel movements in PD patients with constipation [33]. Future studies are warranted to verify and optimize the efficacy of gut microbiota-modulation based strategy against PD. Forth, a certain degree of consistency of microbiota along the gastrointestinal tract has been observed and an individual’s salivary microbiota was found to share some similarity with gut microbiota of the same individual [34]. In addition, the oral bacterial strain is proven to colonize in the gut, which is involved in the disease pathogenesis [35]. This suggests that the oral microbiota should also be considered to understand the “gut-brain axis” [36]. Fifth, although the alterations of gut microbiota in PD patients in comparison with controls have been demonstrated by a series of studies, and several pathways have been proposed to be involved, including the initiation of α-synuclein pathology in the gut, microbial products initiating inflammation and oxidative stress in the brain [30, 37–39], the relationship between gut microbiota and PD still remains to be fully elucidated. This will benefit from better understanding of the molecular basis underlying the “gut-brain axis”. In addition, as the number of studies concerning the relationships between gut microbiota and PD is relatively limited, we can also obtain larger sets of microbiota data to gain implications from the studies on gut microbiota and other neurodegenerative diseases with similar pathogenesis to PD, such as Alzheimer’s disease and amyotrophic lateral sclerosis in future.

In summary, in view of the great potential of gut, oral and nasal microbiota as diagnostically biomarker, and the therapeutic potential of gut microbiota, more well-designed clinical studies recruiting large-scale patients are encouraged on these issues under the condition of steadily increasing prevalence and lack of effective treatment options of PD.

## Data Availability

Not applicable.

## Abbreviations

PD: Parkinson’s disease
SCFAs: Short chain fatty acids
